# Supplementary material for: Cdc7 activates replication checkpoint by phosphorylating the Chk1-binding domain of Claspin in human cells
Source: eLife. 2019 Dec 31;8:e50796. doi: 10.7554/eLife.50796 (PMC6996922; doi:10.7554/eLife.50796)
Supplement: Supplementary file 1. [file elife-50796-supp1.docx]

| **Key Resources Table** | | | | | |
| --- | --- | --- | --- | --- | --- |
| **Reagent type (species) or resource** | **Designation** | **Source or reference** | **Identifiers** | **Additional information** |  |
| Strain, strain background (*Escherichia coli*) | DH5α | TOYOBO | 6557A3 | Competent cells |  |
| Cell line (*Homo-sapiens*) | HeLa | ATCC | CCL-2  RRID:CVCL_0030 |  |  |
| Cell line (*Homo-sapiens*) | 293T | ATCC | ACS-4500  RRID:CVCL_4V93 |  |  |
| Cell line (*Homo-sapiens*) | HCT116 | ATCC | CCL 247  RRID:CVCL_0291 |  |  |
| Cell line (*Homo-sapiens*) | NHDF | ATCC | PCS-201-010  RRID, not available |  |  |
| Cell line (*Homo-sapiens*) | U2OS | ATCC | HTB-96  RRID:CVCL_0042 |  |  |
| Cell line (*Homo-sapiens*) | HCT-15-Luc#1 | JCRB | JCRB1479  RRID:[CVCL_4Y02](https://web.expasy.org/cellosaurus/CVCL_4Y02) |  |  |
| Cell line (*Homo-sapiens*) | NCI-H1975-Luc | JCRB | NIHS0724  RRID:[CVCL_4W96](https://web.expasy.org/cellosaurus/CVCL_4W96) |  |  |
| Cell line (*Homo-sapiens*) | NUGC-3 | JCRB | NIHS0731  RRID:[CVCL_4Y07](https://web.expasy.org/cellosaurus/CVCL_4Y07) |  |  |
| Cell line (*Homo-sapiens*) | HL-60 | JCRB | JCRB0163  RRID:CVCL_2944 |  |  |
| Cell line (*Homo-sapiens*) | 5K-BR-3-Luc | JCRB | NIHS0735  RRID:CVCL_4Y11 |  |  |
| Cell line (*Homo-sapiens*) | KM12-Luc | JCRB | NIHS0647  RRID:CVCL_J258 |  |  |
| Cell line (*Homo-sapiens*) | RPE-1 | From the laboratory of Dr. Eiji Hara (Osaka University) | RRID:CVCL_4388 |  |  |
| Cell line (*Homo-sapiens*) | TIG-3 | From the laboratory of Dr. Eiji Hara (Osaka University) | RRID:CVCL_E939 |  |  |
| Cell line (*Mus- musculus*) | Mouse Embryonic Fibroblasts (MEFs) | In our lab | RRID, not available | Established from E12.5 wild-type mouse embryos |  |
| Cell line (*Mus- musculus*) | *Claspin flox/-* Mouse Embryonic Fibroblasts (MEFs) | In our lab (Yang *et al*. 2016) | RRID, not available | Established from E12.5 *Claspin flox/-* mouse embryos |  |
| Plasmid | pMX-IP | CBL | RTV-014 | retroviral expression vectors |  |
| Plasmid | lentiCRISPR v2 | Addgene | #52961 | lenti expression vectors |  |
| Gene (*Homo-sapiens*) | Cdc7 | https://cansar.icr.ac.uk/cansar/cell-lines/HCT-116/copy_number_variation/chromosome_1/ |  |  |  |
| Antibody | Anti-Chk1(mouse monoclonal antibody) | Santa Cruz Biotechnology | sc-8408 | 1:200) |  |
| Antibody | Anti-Chk1(rabbit polyclonal antibody) | Santa Cruz Biotechnology | sc-7898 | 1:200 dilution for western |  |
| Antibody | Anti-MCM2(goat polyclonal antibody) | Santa Cruz Biotechnology | sc-9839 | 1:200 dilution for western |  |
| Antibody | Anti-MCM2 S53(rabbit polyclonal antibody) | Bethyl | A300-756A | 1:1000 dilution for western |  |
| Antibody | Anti-Cdc7(mouse monoclonal antibody) | MBL | K0070-3 | 1:1000 dilution for western |  |
| Antibody | Anti-Chk1(mouse antibody) | MBL | K0086-3 | 1:1000 dilution for western  5 µg used for immunoprecipitation |  |
| Antibody | Anti-Flag(mouse monoclonal antibody) | MBL | M185-3L | 1:1000 dilution for western |  |
| Antibody | Anti-CK1γ1(rabbit polyclonal antibody) | Biorbyt | orb224102 | 1:2000 dilution for western |  |
| Antibody | Anti-Chk1 S317(rabbit polyclonal antibody) | Cell Signalling | #2341 | 1:1000 dilution for western |  |
| Antibody | FITC-conjugated anti-BrdU antibody(mouse monoclonal antibody) | BD biosciences | 51-33284X | 10 μl for FACS analyses |  |
| Antibody | Anti-tubulin(mouse monoclonal antibody) | Sigma-Aldrich | T5168 | 1:1000 dilution for western |  |
| Antibody | Anti-human Claspin(rabbit polyclonal antibody) | In the lab (Yang *et al*. 2016) |  | 1:1000 dilution for western  5 µl anti-sera for immunoprecipitation  Described in Yang *et al.* 2016 |  |
| Antibody | Anti-mouse Claspin(rabbit polyclonal antibody) | In the lab (Yang *et al.* 2016) |  | 1:500 dilution for western  Described in Yang *et al.* 2016 |  |
| Antibody | Anti-MCM4(rabbit polyclonal antibody) | In the lab (Masai *et al.* 2006) |  | 1:1000 dilution for western  Described in Masai *et al*. 2006 |  |
| Antibody | Anti-MCM4 S6T7(rabbit polyclonal antibody) | In the lab (Masai *et al*. 2006) |  | 1:1000 dilution for western  Described in Masai *et al*. 2006 |  |
| Sequence-based reagent | Claspin FL-F | This paper | PCR primers | ccgctcgagactagtatgacaggcgaggtgggttctg |  |
| Sequence-based reagent | Claspin FL-R | This paper | PCR primers | ctagtctagagctctccaaatatttgaagatgc |  |
| Sequence-based reagent | 897-1100-R | This paper | PCR primers | acctctagactttcttgatttgactctgcagttcc |  |
| Sequence-based reagent | 897-1100-F | This paper | PCR primers | accggctagcgccagtatggatgagaatgcc |  |
| Sequence-based reagent | Cdc7 FL-F | This paper | PCR primers | cagcggccgcggatccatggaggcgtctttgggga |  |
| Sequence-based reagent | Cdc7 FL-R | This paper | PCR primers | ggagaggggcggatcccaagctcatatctttaaaaaatgga |  |
| Sequence-based reagent | siClaspin-sense | This paper | siRNA | uuggccacugauuucaauutt |  |
| Sequence-based reagent | siClaspin-anti-sense | This paper | siRNA | aauugaaaucaguggccaatt |  |
| Sequence-based reagent | siCdc7-sense | This paper | siRNA | gcagucaaagacuguggautt |  |
| Sequence-based reagent | siCdc7-anti-sense | This paper | siRNA | auccacagucuuugacugctt |  |
| Sequence-based reagent | siCK1γ1-sense | This paper | siRNA | ggcaauaagaaagagcaugtt |  |
| Sequence-based reagent | siCK1γ1-anti-sense | This paper | siRNA | caugcucuuucuuauugcctt |  |
| Sequence-based reagent | siGENOME Non-Targeting siRNA Control Pool #1 | Dharmacon | D-001206-13-05 |  |  |
| Peptide, recombinant protein | CKBD-Wild type | Dgpeptides Co. Ltd. |  | ANMDELLDLCTGKFTSQAEKHLPRKSDKKENMEELLNLCSGKFTSQDAST |  |
| Peptide, recombinant protein | CKBD-A | Dgpeptides Co. Ltd. |  | ANMDELLDLCAGKFTSQAEKHLPRKSDKKENMEELLNLCAGKFTSQDAST |  |
| Peptide, recombinant protein | Others-A | Dgpeptides Co. Ltd. |  | ANMDELLDLCAGKFTSQAEKHLPRKSDKKENMEE |  |
| Peptide, recombinant protein | Others-E | Dgpeptides Co. Ltd. |  | ANMDELLDLCTGKFEEQAEKHLPRKEDKKENMEELLNLCSGKFESQDAEE |  |
| Peptide, recombinant protein | NC | BSI Co. Ltd. |  | [Biotin]ENAMDANMDELLDLC[pT]GKFTSQAEKHLPRKSDKKENMEELLNLC[pS]GKFTSQDAST[COOH] |  |
| Peptide, recombinant protein | N | BSI Co. Ltd. |  | [Biotin]DANMDELLDLC[pT]GKFTSQAEK[COOH] |  |
| Peptide, recombinant protein | C | BSI Co. Ltd. |  | [Biotin]KENMEELLNLC[pS]GKFTSQDAS[COOH] |  |
| Peptide, recombinant protein | Cdc7-ASK | Carnabio Co. Ltd. | 05-109 | Purified protein |  |
| Peptide, recombinant protein | Chk1 | Carnabio Co. Ltd. | 02-117 | Purified protein |  |
| Peptide, recombinant protein | CK1γ1 | Carnabio Co. Ltd. | 03-105 | Purified protein |  |
| Peptide, recombinant protein | Cdc7-ASK | In the lab (Masai *et al*. 2000) |  | Cdc7-ASK was purified from Sf9 cells as previously described (Masai *et al*. 2000) |  |
| Commercial assay or kit | In Situ Direct DNA Fragmentation Assay Kit | Abcam | ab66108 | Reagent for cell death assays |  |
| Chemical compound,drug | XL413 | AdooQ Bioscience, Co. Ltd. | BMS-863233 | Chemical Cdc7 inhibitor |  |
| Chemical compound,drug | PEI | Polyscience Co. Ltd. | Cat.24765 | Transfection reagent |  |
| other | anti-Flag M2 affinity beads | SIGMA | A2220 | Mouse monocle antibody conjugated to agarose beads |  |
| other | Benzonase | Amersham Plc. |  | DNA degrading enzyme  10 units⁄ ml |  |
| other | Protease inhibitor-PI tablet | Roche | 4693159001 |  |  |
| other | DAPI stain | Invitrogen | D1306 | Staining of DNA 1 µg/mL |  |
