# Supplementary material for: Cdc7 activates replication checkpoint by phosphorylating the Chk1-binding domain of Claspin in human cells
Source: eLife. 2019 Dec 31;8:e50796. doi: 10.7554/eLife.50796 (PMC6996922; doi:10.7554/eLife.50796)
Supplement: Supplementary file 2. [file elife-50796-supp2.docx]

**Supplementary Material**

**Cdc7 activates replication checkpoint by phosphorylating the Chk1 binding domain of Claspin**

**Chi-Chun Yang^1^, Hiroyuki Kato^1^, Mayumi Shindo^2^ and Hisao Masai*^1^**

**^1^**Department of Genome Medicine, **^2^**Protein Analyses Laboratory, Tokyo Metropolitan Institute of Medical Science, 4-6-1 Kamikitazawa, Setagaya-ku, Tokyo 156-8506, Japan

Running title:

Cdc7 kinase in replication checkpoint activation

*****Correspondence should be addressed to

Hisao Masai

E-mail: masai-hs@igakuken.or.jp

Telephone: +81-3-5316-3231

Fax: +81-3-5316-3145

**Supplementary Table S1**

**Sequences of the oligonucleotides used in this study**

| Name | Sequence (5’->3’) |
| --- | --- |
| Claspin FL-F | ccgctcgagactagtatgacaggcgaggtgggttctg |
| Claspin FL-R | ctagtctagagctctccaaatatttgaagatgc |
| 897-1100-R | acctctagactttcttgatttgactctgcagttcc |
| 897-1100-F | accggctagcgccagtatggatgagaatgcc |
| Cdc7 FL-F | cagcggccgcggatccatggaggcgtctttgggga |
| Cdc7 FL-R | ggagaggggcggatcccaagctcatatctttaaaaaatgga |
| siClaspin-sense | uuggccacugauuucaauutt |
| siClaspin-anti-sense | aauugaaaucaguggccaatt |
| siCdc7-sense | gcagucaaagacuguggautt |
| siCdc7-anti-sense | auccacagucuuugacugctt |
| siCK1γ1-sense | ggcaauaagaaagagcaugtt |
| siCK1γ1-anti-sense | caugcucuuucuuauugcctt |
